# Use of PIT tags to assess individual heterogeneity of laboratory-reared juveniles of the endangered Cumberlandian combshell (*Epioblasma brevidens*) in a mark–recapture study

**DOI:** 10.1002/ece3.1348

**Published:** 2015-02-13

**Authors:** Dan Hua, Yan Jiao, Richard Neves, Jess Jones

**Affiliations:** 1 Department of Fish and Wildlife Conservation, College of Natural Resources, Virginia Polytechnic Institute and State UniversityBlacksburg, Virginia; 2 Freshwater Mollusk Conservation Center, Virginia Polytechnic Institute and State UniversityBlacksburg, Virginia; 3 U.S. Geological Survey, Cooperative Fish and Wildlife Research UnitBlacksburg, Virginia; 4 U.S. Fish and Wildlife Service, Ecological Services OfficeBlacksburg, Virginia

**Keywords:** Bayesian, Cumberlandian combshell (*Epioblasma brevidens*), detection probability, heterogeneity, hierarchic model, mark–recapture, PIT tag, survival rate

## Abstract

The federally endangered Cumberlandian combshell (*Epioblasma brevidens*) was propagated and reared to taggable size (5–10 mm), and released to the Powell River, Tennessee, to augment a relict population. Methodology using passive integrated transponder (PIT) tags on these mussels greatly facilitated the detection process. The overall mean detection probability and survival rate of released individuals reached 97.8 to 98.4% and 99.7 to 99.9% (per month), respectively, during nine successive recapture occasions in the 2-year study period, regardless of seasonality. Nonhierarchical models and hierarchical models incorporating individual and seasonal variations through a Bayesian approach were compared and resulted in similar performance of prediction for detection probability and survival rate of mussels. This is the first study to apply the mark–recapture method to laboratory-reared mussels using PIT tags and stochastic models. Quantitative analyses for individual heterogeneity allowed examination of demographic variance and effects of heterogeneity on population dynamics, although the individual and seasonal variations were small in this study. Our results provide useful information in implementing conservation strategies of this faunal group and a framework for other species or similar studies.

## Introduction

Global diversity of freshwater mussels (Unionoida) is estimated to be 860 species (Graf 2013). Approximately 37% of this fauna occurs in North America, which contains the world's greatest diversity (Neves 2008); 281 species and 16 subspecies (Williams et al. 1993). However, this order of mollusks is globally declining and has become the most imperiled group of animals in North America (Haag & Williams 2014). Of the United States taxa, only 70 species (23.6%) are considered stable, while 213 species (71.7%) are considered imperiled, endangered, threatened, extinct, or of special concern to state or federal agencies (Neves and Williams 1994). Roughly, 30 species are assumed extinct, disappearing in the last 100 years (Neves 2008), and 84 species are listed as federally endangered or threatened (USFWS (U. S. Fish and Wildlife Service) 2013), with that number expected to increase (Shannon et al. 1993). The mussel declines are attributed to habitat degradation and destruction mostly due to water pollution, dams, sedimentation, and urbanization effects (Parmalee and Bogan 1998; Neves 1999, 2008). Without immediate efforts to recover federally protected species in watersheds throughout the country, extinction of additional species is inevitable.

The Powell River is located in eastern Tennessee (TN) and southwestern Virginia (VA) and is a tributary to the Tennessee River system. Historically, the river contained up to 46 species of freshwater mussels (Ortmann 1918; Johnson et al. 2012) and remains one of the most diverse faunas in the United States, with at least 32 extant species. However, overall declines in mussel abundance and species richness have been chronicled, and the mean mussel density (mussels/m^2^) has declined by 63% in this river from 1979 to 2004 (Ahlstedt et al. 2005). Numerous species are nearing extirpation, including the federally endangered Cumberlandian combshell (*Epioblasma brevidens*) (Johnson 2011). This species was listed as endangered under the federal Endangered Species Act of 1973. Globally, it is designated as critically endangered on the IUCN Red List of threatened species. Besides the Powell River, *E. brevidens* has been extirpated from most of its former range in the Cumberland and Tennessee River systems (USFWS (U. S. Fish and Wildlife Service) 2003). Given the high degree of threat and low recovery potential of this species, a recovery plan for *E. brevidens* was prepared and approved by the U.S. Fish and Wildlife Service in 2004 (USFES (U. S. Fish and Wildlife Service) 2004). Recovery plans for endangered species proposed a strategy of propagation and release of young mussels to their natal rivers to augment remnant populations or to reintroduce populations into historic habitats (USFWS (U. S. Fish and Wildlife Service) 2007; Neves 2008).

Monitoring of restored populations is an important and essential approach to evaluate the success of mussel release efforts, which has involved mark–recapture programs. Current methods using numbered glue-on tags (Kjos et al. 1998; Villella et al. 2004; Peterson et al. 2011) or direct curved labels on mussel shells (Hua 2005) are inefficient due to low recapture rate (Villella et al. 2004; Peterson et al. 2011; Rogers 1999). Hence, a reliable and efficient tagging methodology is desirable to effectively recapture released mussels. The passive integrated transponder (PIT) technology was originally developed in the 1980s to relocate animals (Fagerstone and Johns 1987; Schooley et al. 1993) and was rapidly expanded to track activities of a wide range of taxa including amphibians, birds, fish, and mammals (Germano and Williams 1993; Becker and Wendeln 1997; Burns et al. 1997; Zydlewski et al. 2001; Galimberti et al. 2000). As a mark–recapture tool, this novel technique was recently applied to monitor survival of freshwater mussels. Kurth et al. (2007) found that use of PIT tags doubled mussel recapture rates (72–80%) over visual observation (30–47%) at all sites, with a retention rate of 93%. On the basis of previous studies, we developed a protocol to apply electronic PIT tags on laboratory-produced juveniles of *E. brevidens* and evaluated effectiveness of the technique through recapture rates and demographic analysis.

Demographic modeling has been widely developed to estimate parameters of animal life history through capture–recapture protocols (Williams et al. 2002; Pledger et al. 2003). The traditional mark–recapture models often assume homogeneity in animal survival, capture probabilities, and individual variability, which likely introduces bias into model selection and parameter estimation (Pledger et al. 2003), resulting in misunderstanding of life-history traits (Cam et al. 2002) and high variability in small populations (Conner and White 1999). To estimate variability among individuals, models have been developed and constructed to allow individual heterogeneity using computer programs and thus have enabled significant applications for understanding population biology (Pledger et al. 2003). Consequently, stochastic models with individual variation can capture the uncertainty of estimated parameters with that variability incorporated; hence reflecting biological reality to a better degree (Pfister and Stevens 2003; Gimenez and Choquet 2010; McVinish and Pollett 2013). Markov chain Monte Carlo (MCMC) algorithms used in Bayesian statistical inference provide a mathematical framework to circumvent the problem of high-dimensional integrals and allow the likelihood function to be conditional on the unobserved variables in models, simplifying and expediting Bayesian parameter estimation (Gimenez 2008; Paap 2002; Wade 2000).

Effective conservation and restoration strategies of endangered mussel species will require knowledge of population dynamics and predictions of population growth. Our goals were to develop empirical models to estimate changes in mussel population after release to natal rivers, with the following objectives: (1) develop a recapture method to increase mussel recapture rates, (2) develop models incorporating the heterogeneity of individual variations and seasonal changes in survival and detection probabilities for live and dead mussels, and (3) demonstrate applications of stochastic analyses through a Bayesian approach to minimize bias in parameter estimation.

## Methods and Materials

### Juvenile mussels

Juvenile mussels of *E. brevidens* were propagated and cultured at the Freshwater Mollusk Conservation Center, Department of Fish and Wildlife Conservation, Virginia Tech, Blacksburg, VA.

### Tagging methods

Subadult mussels were tagged upon reaching 5 mm in length using bee tags (The Bee Works, Ontario, Canada), and FPN glue-on shellfish tags (Hallprint, Hindmarsh, Australia) for the larger individuals (=10 mm in length). Each bee tag was glued on the mussel umbo, and a FPN shellfish tag was adhered to the external valve using “Loctite super glue”. Bulk PIT tags (TX1411SST, Biomark, Boise, Idaho) were used in this study because of their high radio-frequency identification performance and small size (12.5 × 2.1 mm). Each electronic PIT tag is coded with one of 10 trillion unique codes. The Portable BP antenna and FS2001F-ISO reader (Biomark, Boise, Idaho) were used to detect mussels with attached PIT tags in our study. The PIT tag is activated when the Portable BP antenna reaches its detection area and immediately responds with sound, and the unique digital code is transmitted back to the reader. Mussels were affixed with PIT tags once they grew to subadults, at least 20 mm in length. We developed a tagging method to apply PIT tags on mussels. PIT tags were applied using a 3-step process. Shells were cleaned and dried, PIT tags were attached with cyanoacrylate glue, and tags were embedded with dental cement (Fuji Glass Ionomer Luting Cement, Japan). To minimize any negative effects, the whole process to conduct PIT tagging on each mussel was completed in <2 min and in the shade under field conditions to reduce stress.

### Release and sample

**Figure 1:**
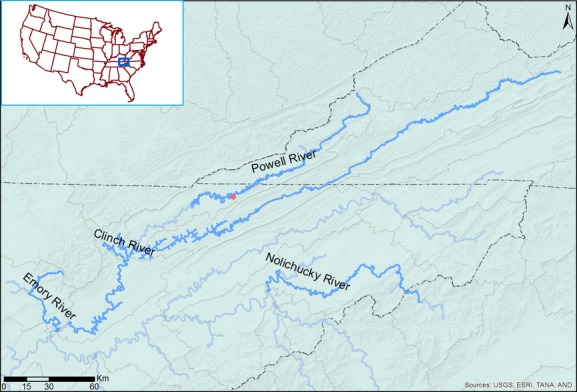
Release of juvenile mussels of *E. brevidens* at Brooks Bridge (36.534879, −83.441762) in the Powell River, Tennessee, USA.

A mark–recapture method was used to monitor released mussels. Subadult mussels of *E. brevidens* were tagged when they reached a minimum size of 20 mm (in length) and released to a site (36.535109, −83.441621) near Brooks Bridge, Powell River, Tennessee (Fig.1). River width at the site is around 30 m. The release site is about 50 m^2^ in area, with 25–40 cm depth of variable substrates, from clay and sand to infrequent cobbles and boulders. Discharge varies seasonally with precipitation levels, typical of rivers. Mussels were released at multiple times, on 1 July 2009, 26 August 2009, 7 October 2009, 25 June 2010, and 11 October 2010 (Table1), respectively, once they reached the taggable size at FMCC or in cages held at the release site. The in situ cage was built from a plastic storage container (13 cm × 38 cm × 53 cm). The lid and bottom of the container was constructed with a sheet of plastic mesh screening (square mesh size of 1.3 cm) to allow water flow. The cage was filled to one-third with substrate collected at the site of release to receive the small juveniles and then was deployed into the substrate. We developed this cage to retain young juveniles (prior to taggable size) to monitor their natural survival and growth rate. Later, this tagged and released group was relocated by PIT tag detector to determine survival, shell length and then returned to their captured locations. Recapture sampling occurred from 1 July 2009 until 11 October 2011, with a total of eight sample periods (Table1). We began sampling 5 m downstream of the release site and moved upstream to 5 m above the release site. Similarly, the sampling area extended 5 m to the left and right margins of the release site. Each PIT tag detected by the portable BP antenna and reader allowed determination whether an individual mussel was live or dead. Located mussels were excavated by snorkeling or view-scopes to record survival and shell length. To increase the likelihood of recapture, we scanned the site three times during each sampling event. More than 95% of mussels were captured during the first pass of scanning, and no mussels were detected during the third scan.

**Table 1:** Release and recapture of laboratory-produced juvenile mussels (*E. brevidens*)

Date	1/7/09	26/8/09	7/10/09	25/6/10	11/10/10	10/5/11	17/8/11	12/10/11
Age (months)	24.5	26.5	28	36.5	40	47	50	52
Number of released mussels	23	28	38	9	1	0	0	0
Number of recaptured live mussels		23	50	88	97	96	95	94
Number of recaptured dead mussels	0	0	0	0	1	0	0	0

### Modeling

An individual model was designed and used in this study with the following assumptions:

- Labeled subadult mussels retained their tags throughout the study. PIT tags were detectable with certain rate, without negative influence to their survival.
- Variation of survival rates among seasons followed a certain random distribution.
- Sampling protocol and study area were constant.

#### Data of observations and models

The capture history of individuals was recorded as a sequence of 0s and 1s to indicate that each individual was seen or not during the sampling periods. For example, the history (0 1 1 0 1 1 0 1 0) indicates that the tagged mussel with a unique code was captured for the first time at sampling occasions 2 and 3, not capture at time 4, captured again at times 5, 6, and 8, but not at times 7 and 9. Use of PIT tags allowed us to recapture live mussels and dead ones (shells). Two data sets, including capture history of live and dead individuals, were used for analyses.

Formulation for Model 1 as follows:

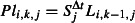


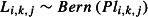


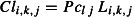


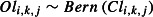


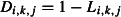


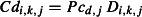
1

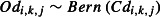
where *i* represents the *i*th individual, *j* represents the season of sampling occasions (*j* = 1 denotes summer from June to October, when mussels grow rapidly, *j* = 2 denotes winter from October to May, a slow growing season), *k* represents the *k*th capture occasions (*t *= 0, 1, 2…7, *t *= 0 denotes as the time of mussel release and the 0th capture), *j* represents the season of sampling occasions, and Δ*t* represents time (month) between adjacent capture occasions. *L*_*i,k,j*_ represents the live statue of a individual mussel at the *k*th capture occasions of the season *j*. *S*_*j*_ is the monthly survival rate of *E. brevidens* in the summer or winter, and its prior is assumed to follow a uniform distribution between 0 and 1. *Pl*_*i,k,j*_ denotes the probability of a live individual at the capture occasion *K*th that depends on its survival status at the (*k* − 1)th recapture occasion and survival rate *S*_*j*_. *Cl*_*i,k,j*_ denotes probability of recapturing a live mussel based on recapture rate *Pc*_*l j*_ and its survival status at the *k*th capture occasion. *Ol*_*i,k,j*_ is the observation of live individual that follows Bernoulli distribution. *D*_*i,k,j*_ denotes as the status of a dead individual. *Cd*_*i,k,j*_ denotes probability of recapturing a dead mussel based on recapture rate *Pc*_*d j*_ and its death status at the *k*th capture occasion. *Od*_*i,k,j*_ is the observation of dead individual that follows Bernoulli distribution. *S*_*j*_, *Pc*_*l j*_ and *Pc*_*d j*_ represent population characteristics and were assumed to follow uniform distributions between 0 and 1.

Besides the above model, the Model 2 was constructed assuming the seasonal variations only in detection probability of dead mussels (*Pc*_*d j*_), but ignoring them in survival rate (*S*) and detection probability of live mussels (*Pc*_*l*_); Model 3 was constructed ignoring seasonal variation in all *S*,*Pc*_*d*_, and *Pc*_*l*_; Model 4 only included the parameters of *S* and *Pc*_*l*_ without seasonal variation and records for dead mussels due to limited observations; Models 1-1, 2-1, 3-1, and 4-1 were constructed hierarchically considering the individual variations in the above correspondent models.

Formulation for Model 1-1:

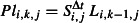


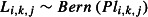


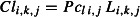


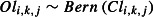


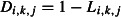


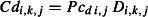


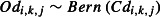


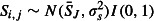


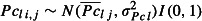


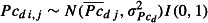
2where *S*_*i,j*_, *Pc*_*l i,j*_, and *Pc*_*d i,j*_ represent population characteristics and were assumed to follow normal distributions 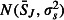, 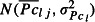, and 
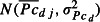
, respectively. The 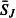, 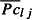, and 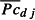 represent mean values of population characteristics of *S*_*i,j*_, *Pc*_*l i,j*_, and *Pc*_*d i,j*_ and among individuals and were assumed to follow uniform distributions between 0 and 1 because uniform prior distributions for variance outperformed in hierarchical models (Gelman 2006). *I* denotes the boundary of the distribution in WinBUGS.

#### Priors

Multilevel priors incorporating seasonal variations and individual differences were tested to estimate survival and recapture rates and their associated uncertainty. The Bayesian approach was used to calculate a posterior distribution from the observed data and multilevel prior distribution.

WinBUGS is a numerically intensive software package for the Bayesian analysis of complex statistical models to implement Markov chain Monte Carlo (MCMC) methods (Spiegelhalter et al. 1996). We used WinBUGS software associated with three Markov chains to determine the convergence of the posterior distribution by monitoring the trace plot, diagnosing the autocorrelation and Gelman and Rubin statistics (Spiegelhalter et al. 2004; Jiao et al. 2008, 2009). A proper burn-in iteration and thinning interval were determined based on the convergence criteria (Jiao et al. 2008). The Bayesian inference was generated from the samples of random draws from the posterior distribution after the three chains converged.

#### Model goodness of fit

Deviance information criterion (DIC) was used to determine the model goodness of fit for the Bayesian models and formulated as:

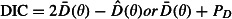


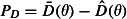
3

where D is the deviance to measure the predicted goodness of fit for all eight models, *P*_*D*_ is the effective number of parameters in a Bayesian model, 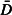 is the posterior mean of the deviance, and 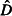 is the deviance of the posterior mean. The DIC is particularly applied in Bayesian model selection using MCMC simulation to determine the posterior distributions of the models (Spiegelhalter et al. 2002). Model 4 and Model 4-1 were constructed by excluding the data for detection probability of dead mussels. The other six models were produced using the data including both detection probabilities of live and dead mussels. Thence, models were compared between Model 4 and Model 4-1, and among the other six models. Models were evaluated based on the DIC values, which model with the lowest DIC made the best prediction. Models within 5 DIC units of the “best” model received the best consideration. Those within 5–10 DIC units of the “best” model were considered substantially less well supported. Models more than 10 DIC units from the “best” model were definitely excluded (Spiegelhalter et al. 2002; Jiao et al. 2009).

## Results

**Figure 2:**
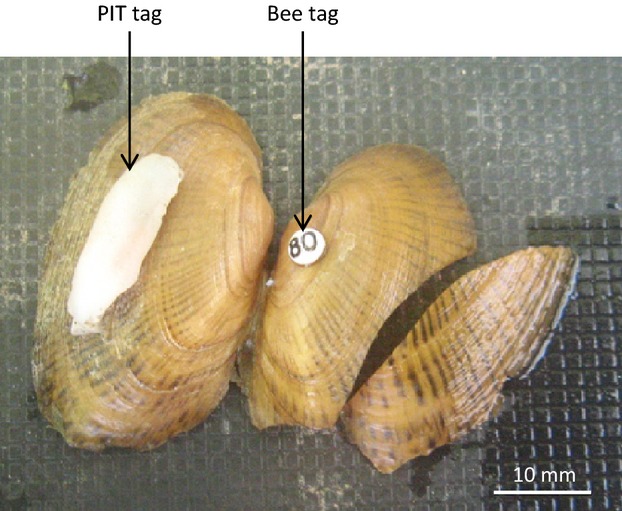
Dead mussel located and recaptured using PIT tag protocol.

During the 2-year sampling period, 100% of recaptured mussels retained their PIT tags. A dead mussel was recaptured from the substrate at an approximate depth of 35 cm by the affixed PIT tag on one piece of broken shell (Fig.2). Vertical and horizontal movements of released mussels were observed as they positioned themselves from surface to a depth of 35 cm in the substrate, with seasonal variations. Mussels were detected and recaptured whether they burrowed in sand, clay, gravel, and even beneath cobbles and boulders.

Estimated mean survival rate of released mussels (*S*) among all models reached 0.997 to 0.999/month during the 2-year period, indicating that the release site in Powell River is suitable for this endangered species. The mean detection probability for live mussels (*Pc*_*l*_) ranged from 0.978 to 0.984 (Table2). Seasonal variation in *Pc*_*l*_ and *S* was not detected. The mean detection probability for dead mussels showed slight seasonal variations; *Pc*_*d*_ value ranged from 0.373 to 0.438 in summer and from 0.339 to 0.438 in winter with a relatively high standard deviation ranged from 0.155 to 0.199 in summer and from 0.169 to 0.243 in winter.

**Table 2:** Estimated survival rate (monthly) and detection probability of released mussels in the eight models. The *j* represents the season of sampling occasions (*j* = 1 denotes summer; *j* = 2 denotes winter). The *S*_*j*_ is the monthly survival rate of *E. brevidens* in the summer or winter; *Pc*_*l j*_ and *Pc*_*d j*_ denote the recapture rate of live and dead mussel in the summer or winter, respectively. The 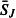, 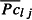, and 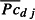 represent mean values of monthly survival, the recapture rates of live and dead mussel among individuals in the summer or winter, respectively. The 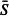, 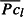, and 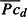 are mean values of monthly survival, the recapture rates of live and dead mussel among individuals without seasonal variations. The *σ*_*s*_, 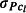, and 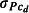 are standard deviations of 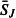, 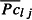, and 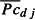 in Models 1-1 and 2-1, and are the standard deviations of 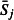, 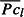, and 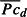 in Models 3-1 and 4-1. The seasonal variations among above standard deviations were not incorporated due to their extremely small values. The *S*,*Pc*_*l*_, *Pc*_*d*_ represent the monthly survival rate and recapture rate for live and dead mussels without seasonal variations

Model	Parameter	Summer		Winter		No seasonal variation	
		Mean	SD	Mean	SD	Mean	SD
Model 1	*S*_*j*_	0.997	0.002	0.999	0.001	–	–
	*Pc*_*l j*_	0.981	0.008	0.984	0.009	–	–
	*Pc*_*d j*_	0.373	0.198	0.343	0.243	–	–
Model 1-1	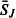	0.997	0.002	0.998	0.001	–	–
	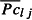	0.978	0.008	0.978	0.010	–	–
	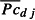	0.428	0.156	0.438	0.174	–	–
	*σ*_*s*_	3.34e^−7^	4.43e^−7^	3.34e^−7^	4.43e^−7^	–	–
	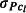	2.43e^−5^	2.64e^−5^	2.43e^−5^	2.64e^−5^	–	–
	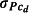	6.48e^−3^	5.77e^−3^	6.48e^−3^	5.77e^−3^	–	–
Model 2	*S*	–	–	–	–	0.999	0.001
	*Pc*_*l*_	–	–	–	–	0.984	0.006
	*Pc*_*d j*_	0.382	0.199	0.339	0.239	–	–
Model 2-1	*S*	–	–	–	–	0.999	0.001
	*Pc*_*l*_	–	–	–	–	0.984	0.006
	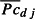	–	–	–	–	0.437	0.169
	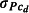	–	–	–	–	6.76e^−3^	5.85e^−3^
Model 3	*S*	–	–	–	–	0.999	0.001
	*Pc*_*l*_	–	–	–	–	0.984	0.006
	*Pc*_*d*_	–	–	–	–	0.321	0.177
Model 3-1	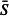	–	–	–	–	0.998	0.001
	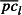	–	–	–	–	0.980	0.007
	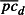	–	–	–	–	0.423	0.133
	*σ*_*s*_	–	–	–	–	5.75e^−7^	7.62e^−7^
	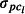	–	–	–	–	4.45e^−5^	4.60e^−5^
	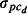	–	–	–	–	1.44e^−2^	1.13e^−2^
Model 4	*S*	–	–	–	–	0.999	0.001
	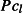	–	–	–	–	0.984	0.006
Model 4-1	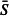	–	–	–	–	0.997	0.001
	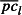	–	–	–	–	0.979	0.007
	*σ*_*s*_	–	–	–	–	7.88e^−7^	1.03e-6
	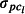	–	–	–	–	4.92e^−5^	5.16e-5

**Figure 3:**
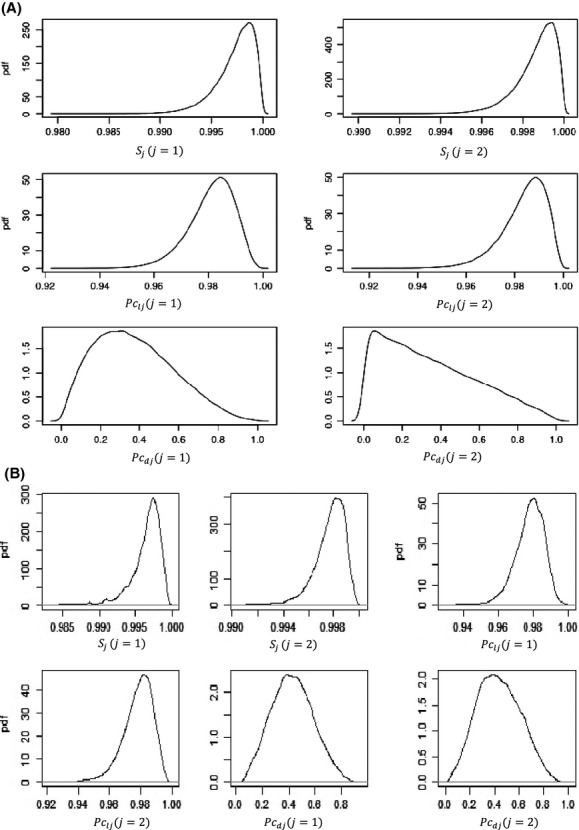
Posterior density function of parameters in models 1 and 1-1. *S*_*j*_, *Pc*_*ij*_, and *Pc*_*dj*_ represent monthly survival rate, probabilities of recapture rates for live and dead *E. brevidens* (*j* = 1 denotes summer, *j* = 2 denotes winter). (A) (Model 1). (B) (Model 1-1).

The distributions of posterior density function (pdf) for parameters *S*,*Pc*_*l*_, and *Pc*_*d*_ are illustrated in Fig.3. The pdf of *S* and *Pc*_*l*_ exhibited a reasonable symmetric curve that approaches a normal distribution; however, the pdf of detection probability for dead mussels was apparently skewed especially in winter, in those models incorporating individual variations (Fig.3A). Variations among individuals in *S* (<10^−5^) and *Pc*_*l*_ (<10^−3^) were very small, only exhibited in *Pc*_*d*_ with a noticeable value (Fig.3B).

In our study, the hierarchical model 4-1, that estimated survival and detection probability of live mussels incorporating individual variations, exhibited a slightly lower DIC value (69.05) compared to the nonhierarchical model 4 (70.22), a difference of 0.17. Among the other six models, the lowest DIC (76.29) identified Model 3 with the best performance. However, the maximum difference in DIC values among the six models was 3.37 units (Table3). Therefore, the overall differences in these six models are not significant. These results reflect the small estimated standard deviation from seasonal and individual variations in models 1-2, 2-2, 3-2, and 4-2. In this study, results showed that hierarchical models incorporating individual variations and nonhierarchical models performed similarly.

**Table 3:** Comparison of DIC values among the eight models (survival rate is per month). The *j* represents the season of sampling occasions (*j* = 1 denotes summer; *j* = 2 denotes winter; *j*_*1*_ = *j*_*2*_ when parameters ignoring seasonal variations). The *S*_*j*_ is the monthly survival rate of *E. brevidens* in the summer or winter; *Pc*_*l j*_ and *Pc*_*d j*_ denote the recapture rate of live and dead mussel in the summer or winter, respectively

Model	All Parameters	Parameters incorporating seasonal variations	Parameters ignoring seasonal variation	Parameters incorporating individual variations	DIC
1	*S*_*j*_, *Pc*_*l j*_, *Pc*_*d j*_	*S*_*j*_, *Pc*_*l j*_, *Pc*_*d j*_	–	–	76.72
1-1	*S*_*j*_, *Pc*_*l j*_, *Pc*_*d j*_	*S*_*j*_, *Pc*_*l j*_, *Pc*_*d j*_	–	*S*_*j*_, *Pc*_*l j*_, *Pc*_*d j*_	79.66
2	*S*_*j*_, *Pc*_*l j*_, *Pc*_*d j*_	*Pc*_*d j*_	*S*_*j*_, *Pc*_*l j*_	–	77.04
2-2	*S*_*j*_, *Pc*_*l j*_, *Pc*_*d j*_	*Pc*_*d j*_	*S*_*j*_, *Pc*_*l j*_	*Pc*_*d j*_	77.94
3	*S*_*j*_, *Pc*_*l j*_, *Pc*_*d j*_	–	*S*_*j*_, *Pc*_*l j*_, *Pc*_*d j*_	–	76.29
3-3	*S*_*j*_, *Pc*_*l j*_, *Pc*_*d j*_	–	*S*_*j*_, *Pc*_*l j*_, *Pc*_*d j*_	*S*_*j*_, *Pc*_*l j*_, *Pc*_*d j*_	78.05
4	*S*_*j*_, *Pc*_*l j*_	–	*S*_*j*_, *Pc*_*l j*_	–	70.22
4-4	*S*_*j*_, *Pc*_*l j*_	–	*S*_*j*_, *Pc*_*l j*_	*S*_*j*_, *Pc*_*l j*_	69.05

## Discussion

Use of quadrats or transects have been applied for quantitative and qualitative mussel sampling (Smith et al. 2001; Strayer and Smith 2003; Ahlstedt et al. 2005). However, these traditional survey methods are inadequate to monitor population changes of rare species of mussels (Johnson 2011). Even for nonlisted mussel species, recapture rates for *Elliptio complanata*,*E. fisheriana,* and *Lampsilis cariosa* were only 3% to 19% during a 2-year mark–recapture study (Villella et al. 2004). Low capture probability results in less precision in estimation of survival rate because that rate is estimated based on the probability of detecting individuals during subsequent sampling occasions (Burham et al. 1987). The standardized protocol for mussel surveys is by visual observation and excavation of substrates. However, the vertical movement and horizontal dispersion of specimens often result in low catchability (Villella et al. 2004). Mark–recapture of *E. brevidens* using PIT technology in our study reached a high detection probability of 98%. The electronic tags detected through a portable BP antenna and reader greatly facilitated the relocation and identification of released individuals.

Kurth et al. (2007) stated that the noncaptured mussels (20–28%) in their study might be due to loss of PIT tags. We noted that tagging method directly influenced the tag retention rates. High recapture rate (=98%) in our study benefited from a proper tagging method. Prior to tagging, the cleaning and drying of the shell surface and prefixing of a glass-encapsulated PIT tag onto the valve using Loctite super glue are critical to the process. Viscosity of the dental cement mixture is also critical. A thin mixture could be eroded by mussel movements, and swift currents can cause PIT tags to be exposed, making them vulnerable to detachment from mussels. A thick cement mixture dries too quickly to envelop the entire PIT tag. We have tagged mussels of multiple species and reared them in the laboratory for many years, and overall survival rates of nearly 100% support the PIT tagging protocol in this study. Our results showed a high recapture probability and survival rate over 2 years, indicating an effective protocol using the described PIT tagging method. However, inappropriate handling methods can cause PIT tag loss or mussel stress. Wilsona et al. (2011) tested mussel behavior in an experiment of mussels with and without PIT tags, and indicted that mussel-burrowing activities were influenced by the additional weight of PIT tags. However, their results did not show significant differences in mussel activity, burrowing ability, burrowing time, and burrowing rate index between the two treatments. Conversely, mussels had a delayed response seemingly attributed to the long process of tagging (40 min) and impact from ethanol used in their study. Ethanol is used to preserve animal tissues and should be used with caution in tagging live mussels. The other concern of PIT tags is the potential of greater predation caused by visibility of white cement. Our released mussels were implanted into the substrates to eliminate this concern. Observations indicated that the coating cement became dark brown after a few months in the river.

The traditional mark–recapture models often assume the homogeneity in animal survival, capture probabilities, and individual variability, which can bias model selection and parameter estimation (Pledger et al. 2003). Computing resolution associated with mathematical development allows for analyzing the heterogeneity and constructing individual models on capture–recapture of animals. Heterogeneous models have been extended to detect the capture probabilities, survival rates, capture probability, population size, birth rates, lifetime growth in body mass, or variability in individual disease status (Pledger and Schwarz 2002; Pledger et al. 2003; Catchpole et al. 2001; Schofield and Barker 2011). We applied individual models to detect heterogeneity in survival rates, and detection probability for live and dead mussels associated with seasonal changes in the Powell River. In most previous studies, the detection probability component for dead mussels was ignored due to limitation of shell recovery. Use of PIT tags also resolves the problem of low recapture rates and associated bias in estimating parameters. Catchpole et al. (2001) modeled the recovery rates of dead abalone through visual searching for shells, and the recovery rates varied from 0.05 to 0.56, influenced by animal visibility and the experience of divers. The detection probabilities of dead mussels in our study were relatively stable, ranged from 0.339 to 0.382, with associated standard errors at 0.199 to 0.239. The high standard errors might be due to the low mortality rate as only one mussel was collected dead and recovered by the PIT detector, excavated from a substrate depth of approximately 35 cm. Results showed that the detection probability for dead mussels differed slightly in summer compared to winter, while other parameters exhibited little difference from season to season. Use of the PIT method can significantly increase detection probabilities for both live and dead animals, regardless of visibility and experience of operators during the sampling process.

Capture–recapture models are now widely used in population biology with the advent of various computing programs; MARK is one of the most commonly used. However, the limitation of this program is that it does not incorporate individual effects, although it can implement a simple MCMC algorithm (White and Burnham 1999; Gimenez and Choquet 2010). Bayesian theory has greatly increased in applicability in population ecology (Gimenez 2008), and allows individual random effects to be modeled (Gimenez and Choquet 2010), particularly in conservation biology with alternative standard statistical procedures (Wade 2000). However, the Bayesian approach does require programming skills and could be time-consuming to process complex models because it uses extensive MCMC simulation to implement the program. We used WinBUGS (14) in R to generate three chains of length 100,000 (with the first 5000 as burn-in) for nonhierarchical models from 1 to 4, where individual variations were not considered. However, we had to increase the iterations to 10,000,000 (with the first 50,000 as burn-in) to make three chains converged for models of 1-1, 2-1, 3-1, and 4-1 along with individual characteristics. We applied these advantages in our individual models to detect the uncertainty of capture and survival rates associated with seasonal changes.

Seasonal variations in detection probability and survival rates of mussels occur in traditional survey methods through surface visual observation and excavation of substrates (Villella et al. 2004). We developed Model 1 to incorporate seasonal variations in the parameters of survival rate (*S*), detection probabilities for live (*Pc*_*l*_) and dead mussels (*Pc*_*d*_), but found that seasonal variations only existed in *Pc*_*d*_, not in *S* and *Pc*_*l*_. Hence, we applied Model 2 with the seasonal variations in *Pc*_*d*_ but not in others, while Model 3 did not incorporate seasonal variations in all parameters. The standard deviation of *Pc*_*d*_ decreased in Model 3 compared to Models 1 and 2; however, it was still as high as 55.1% of mean value of *Pc*_*d*_.

Royle (2008) and Gimenez and Choquet (2010) indicated that models had the best performance which incorporated random effects. We developed three corresponding hierarchical models (1-1, 2-1, and 3-1) incorporating individual variations. The standard deviations of all parameters in hierarchical models were reduced compared to their nonhierarchical models, especially in *Pc*_*d*_. The distribution of posterior density function for parameters *S*,*Pc*_*l*_, and *Pc*_*d*_ exhibited a reasonable symmetric curve (close to normal distribution), including the pdf of detection probability for dead mussels in winter, while it was skewed (mean is not near the center) in those nonhierarchical models. The standard deviation of *Pc*_*d*_ in Model 3-1 decreased 24.9% compared to it in Model 3; however, it was still as high as 32.4% of mean value of *Pc*_*d*_. In accordance with this result, we reconstructed Model 4 to ignore *Pc*_*d*_ and Model 4-1to incorporate individual variations. The results showed that *Pc*_*l*_ and *S* agreed with those in other models. We found that heterogeneity of seasonal variations apparently occurred in the recapture of dead mussels but not in live mussels. Heterogeneity was not significant in the recapture of live mussels, likely due to the similarity among mussels because all derived from the same cohort of propagated mussels and the high recapture rate using the PIT tag method that reduced variations among detected samples. Pit tag methodology allowed detection of almost all tagged individuals, with equal probability of detection whether in summer or winter. Notwithstanding, the heterogeneity in recapture of dead mussels in this study might derive from low mortality because only one dead mussel was recaptured over 2 years.

The Deviance information criterion (DIC) developed by Spiegelhalter et al. (1998) is one of several methods for comparing Bayesian models. Recently, it has been widely incorporated into WinBUGS and OpenBUGS to implement MCMC simulation. Note that the model with the smallest DIC indicates the estimated model with the best prediction, and models within 5 DIC units of the “best” model need to be reported (Spiegelhalter et al. 2002; Jiao et al. 2009). Hence, model 3 is the best model with the minimum DIC value (76.29). Comparing other DIC values to that of model 3, the other five models (Models 1, 2, Models 1-1, 2-1, and 3-1) also performed well because the differences of DIC values were within five units. Similarly, the DIC difference between model 4 (70.22) and model 4-1 (69.05) is <1 unit. Royle (2008) and Gimenez and Choquet (2010) indicated that models using random effects had the better performance with lowest DIC values. In our study, the skewed distributions of posterior density function for *Pc*_*d*_ in models (1, 2, and 3) were adjusted to an asymptotically normal curve in their corresponding hierarchical models (1-1, 1-2, and 1-3), although the hierarchical models incorporating heterogeneity did not outperform those models without individual and seasonal variations. That is due to the small variation in seasons and individuals, with insignificant impacts on the DIC values. Hence, they are considered in this study. It seems to be explained reasonably well by the negligible value of *σ*_*s*_ and 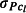 that the similarity of life-history traits occurs in the same cohort of laboratory-produced mussels. Model complexity by adding individual variations might not reveal the advantage of hierarchical models incorporating individual variations in our study. Seasonal variations did not affect survival rates of released mussels, and the PIT tag method provides an equal opportunity for each individual to be captured and to adjust the accuracy of mussel survival rates. Explanations of the heterogeneity in survival rate and detection probability among seasonal and individual variations are still valuable in answering questions of our objectives and providing the framework for other species or similar studies.

We conclude that the mark–recapture model using PIT tags is highly efficient and advances our ability to monitor the restoration of this and other endangered species in rivers. High mortality was only exhibited in the early life stage. Laboratory-reared mussels can exhibit high survival rates where released into suitable habitat. Our methods and results provide optimism for the recovery of this faunal group and can be applied to other faunal groups that are difficult to collect and monitor for conservation and restoration.
