# Supplementary material for: Use of PIT tags to assess individual heterogeneity of laboratory-reared juveniles of the endangered Cumberlandian combshell (Epioblasma brevidens) in a mark–recapture study
Source: Ecol Evol. 2015 Feb 13;5(5):1076–87. doi: 10.1002/ece3.1348 (PMC4364822; doi:10.1002/ece3.1348)
Supplement: Supplementary file 1 [file ece30005-1076-sd1.docx]

Appendix


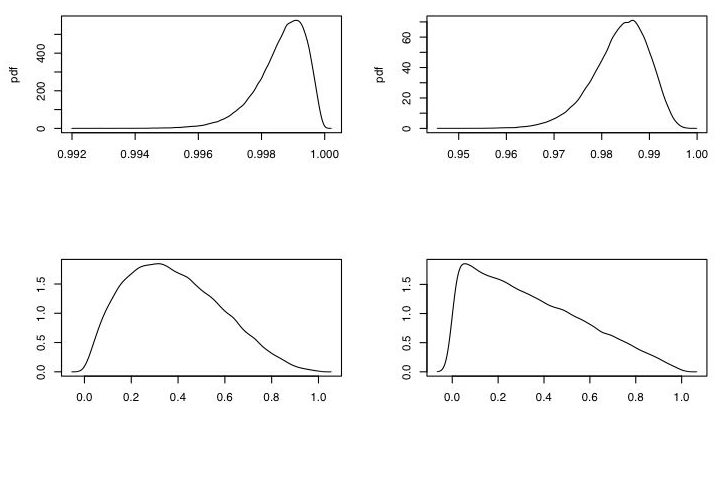

$$S$$

$${Pc}_{l}$$

$${Pc}_{dj}(j=1)$$

$${Pc}_{dj}(j=2)$$

*Model 2*


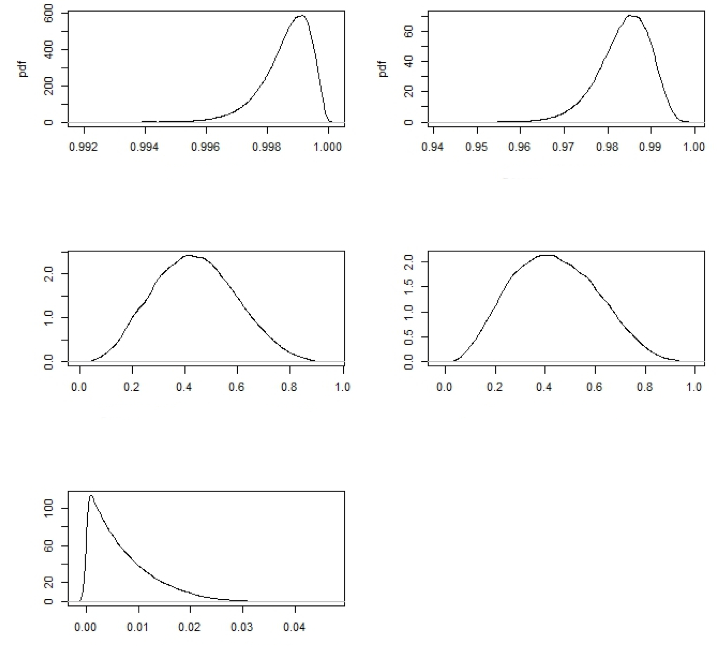

$$S$$

$${Pc}_{l}$$

$${Pc}_{dj}(j=2)$$

$${Pc}_{dj}(j=1)$$

$$\sigma_{{Pc}_{d}}$$

*Model 2-1*


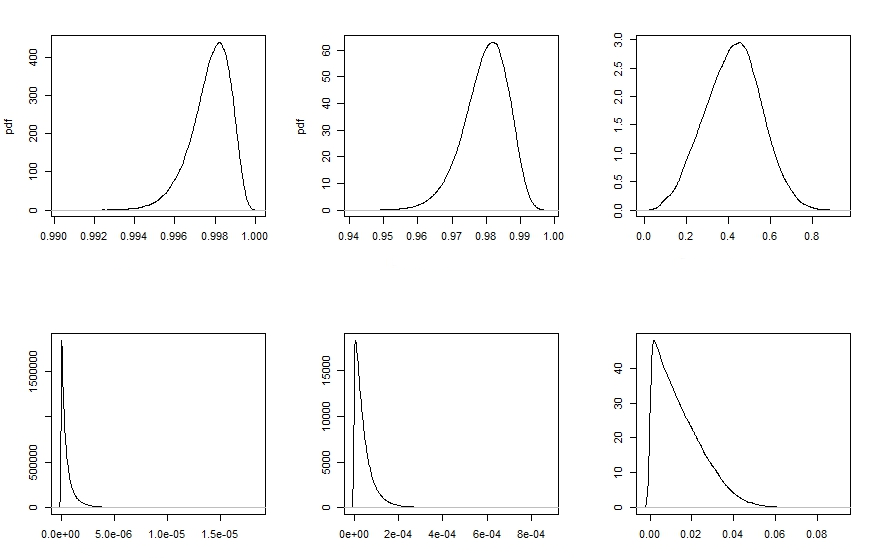

$$S$$

$${Pc}_{l}$$

$${Pc}_{d}$$

$$\sigma_{s}$$

$$\sigma_{{Pc}_{l}}$$

$$\sigma_{{Pc}_{d}}$$

*Model 3-1*


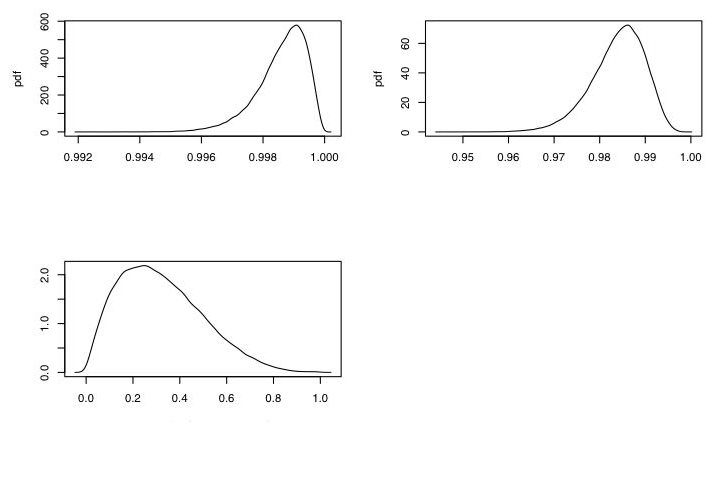

$$S$$

$${Pc}_{l}$$

$${Pc}_{d}$$

*Model 3*


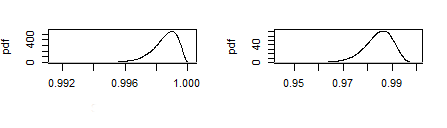

$$S$$

$${Pc}_{l}$$

*Model 4*


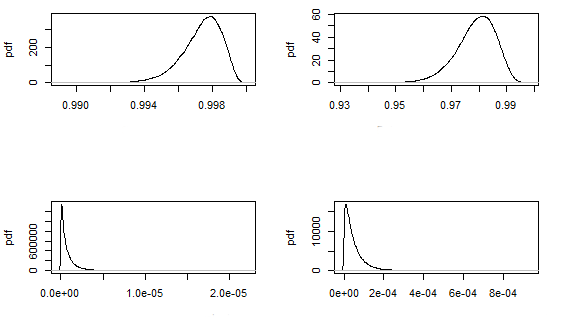

$${Pc}_{l}$$

$$S$$

$$\sigma_{s}$$

$$\sigma_{{Pc}_{l}}$$

*Model 4-1*

Posterior density function of parameters in the other 6 models. $S$and ${Pc}_{l}$ represent the monthly survival rate and probabilities of recapture rates for live *E. brevidens*, ${Pc}_{dj}$ represents the probability of recapture rate for the dead mussel (*j* = 1 denotes summer, *j* = 2 denotes winter).
